# TAL-CGBR: A class-guided boundary regression approach for Temporal Action Localization

**DOI:** 10.1371/journal.pone.0360160

**Published:** 2026-10-12

**Authors:** Jinling Wei, Haolong Chai, Wenwu Hou, Hongwen Yuan, Xiao Huang, Sufen Huang

**Affiliations:** 1 School of Computer and Computing Science, Hangzhou City University, Hangzhou, China; 2 College of Computer Science and Technology, Zhejiang University, Hangzhou, China; 3 Department of Physical Education and Aesthetic Education, Hangzhou City University, Hangzhou, China; University of Electronic Science and Technology of China, CHINA

## Abstract

Temporal Action Localization (TAL) aims to identify all action categories along with their precise temporal boundaries in untrimmed videos. It serves as a fundamental task supporting various downstream applications, including intelligent video editing, automatic summarization, anomaly detection, and human–computer interaction. Despite significant progress, TAL in real-world scenarios remains challenging due to two critical factors: substantial intra-class variations in action duration, ranging from a few seconds to several minutes, and severe degradation of boundary localization accuracy caused by variations in illumination, background clutter, and camera viewpoints. To address these issues, this paper presents a Temporal Action Localization with Class-Guided Boundary Regression (TAL-CGBR) framework, which incorporates semantic information from the classification branch into the regression network to enhance boundary precision. In addition, a window-based attention mechanism and a temporal hierarchical feature structure are introduced to improve computational efficiency while maintaining high localization accuracy. Experimental evaluations on benchmark datasets demonstrate that the proposed TAL-CGBR achieves competitive performance, attaining 68.5% average mAP on THUMOS14 and 36.7% average mAP on ActivityNet v1.3, ranking second among recent methods and demonstrating a favorable balance between accuracy and computational efficiency.

## 1. Introduction

In recent years, substantial progress has been achieved in the field of video understanding, with Temporal Action Localization (TAL) emerging as one of its core tasks [1,2]. TAL aims to determine when and what actions occur in untrimmed videos [2]. This technology has been widely applied to various domains, including intelligent video editing, pedestrian detection for autonomous driving, security surveillance, and human–computer interaction. These applications impose stringent requirements on both the precision and robustness of action boundary localization [3].

Early research primarily focused on manually trimmed short video clips, which typically contain simple scenes and only a single action category. As a result, existing methods are able to To address these issues, this paper presents a Temporal Action Localization with Class-Guided Boundary Regression (TAL-CGBR) framework, which jointly detects action boundaries and categories in untrimmed long videos while maintaining a balance between localization precision and computational efficiency [4–10]. To mitigate the limitations of existing attention-based methods—particularly ActionFormer, which adopts local window attention and multi-scale features but trains classification and regression branches independently—we propose a Class-Guided Boundary Regressor (CGBR). Unlike prior works that treat classification and regression as separate tasks, CGBR explicitly transfers intermediate semantic features from the classification branch to the regression network, providing category-level guidance to refine boundary predictions. This cross-branch semantic fusion is, to the best of our knowledge, the first attempt to leverage classification semantics for boundary regression in TAL. Additionally, while ActionFormer employs a computationally heavy transformer decoder, we introduce a lightweight Temporal Context Module (TCM) that achieves superior performance with fewer parameters.

The main contributions of this paper are summarized as follows:

(1) A Class-Guided Boundary Regressor (CGBR) is designed to incorporate classification semantics into the regression process, effectively enhancing boundary localization accuracy;
(2) A window-based attention mechanism and a temporal hierarchical feature structure are introduced to improve detection efficiency and robustness across varying action durations;
(3) The proposed TAL-CGBR achieves 68.5% average mAP on THUMOS14 and 36.7% average mAP on ActivityNet v1.3, achieving competitive performance against recent state‑of‑the‑art methods, with average mAP of 68.5% on THUMOS14 and 36.7% on ActivityNet v1.3

In summary, while our framework shares certain high-level design choices with ActionFormer [4]—such as local window attention and multi-scale features—the core novelty of our work lies in the CGBR, which introduces semantic guidance into boundary regression. This cross-branch interaction addresses a fundamental limitation of prior works and, as demonstrated in our ablation study, leads to consistent improvements in boundary precision. Moreover, our lightweight TCM design achieves better performance with lower computational cost, and our extensive evaluation across third-person and first-person datasets further validates the robustness and generalization of our approach.

## 2. Materials and methods

**Related works:** Current temporal action localization (TAL) methods can generally be categorized into two main classes according to their dependence on pre-generated temporal proposals.

### 2.1. Two-stage methods

Two-stage methods, represented by VSGN are inspired by the proposal–classification paradigm in image object detection, following the workflow of “proposal generation followed by refined classification and regression” [11]. Specifically, these approaches first generate a set of coarse temporal candidate segments—time intervals that may contain actions—and subsequently perform two tasks for each segment: identifying the action category and refining the start and end boundaries. Although two-stage methods typically achieve high detection accuracy, they suffer from several inherent drawbacks. The generation and refinement of a large number of proposals substantially increase computational complexity, and maintaining high-quality proposals requires carefully designed components, such as specialized generation algorithms, task-specific loss functions, and complex two-branch architectures [12]. Furthermore, the discrepancy in feature distributions between the proposal generation and localization stages may introduce cumulative errors, ultimately degrading boundary precision.

### 2.2. Single-stage methods

The second class, exemplified by Actionformer, employs a single-stage design that directly predicts both the action category and its temporal boundaries within a single inference process, thereby removing the dependency on proposal generation [4]. Single-stage approaches generally offer improved computational efficiency compared with two-stage methods. Recently, Transformer-based frameworks have achieved progress in this category by leveraging the strong sequence-modeling capability of self-attention mechanisms to capture long-range temporal dependencies and complex action dynamics [4–6]. Consequently, single-stage Transformer-based models currently dominate TAL performance benchmarks. The method proposed in this paper belongs to this category and extends prior Transformer-based approaches with improved efficiency and boundary precision.

### 2.3 Transformer-based TAL methods

Although Transformer networks have demonstrated strong representational capacity, their direct adaptation to TAL remains non-trivial. Wang et al. employed a standard Transformer module to model long-term dependencies for boundary prediction, enhancing boundary confidence estimation by learning correlations among temporal segments [13]. However, their approach still relied on manually predefined anchor boxes, limiting adaptability to varying action durations. Tan et al. replaced the standard Transformer encoder with a boundary attention module to better capture temporal context [14]; however, their architecture required three separately trained branch heads, leading to increased model complexity. Moreover, these methods focused primarily on boundary regression while neglecting the joint modeling of action categories and boundaries.

Liu et al., inspired by DETR, introduced an end-to-end Transformer for TAL, but its self-attention mechanism was less effective in capturing short-term dependencies, resulting in suboptimal recognition of short-duration actions [5,15]. Zhang et al. enhanced temporal dependency modeling via a temporal feature pyramid, yet applying self-attention at every feature level substantially increased computational costs [4]. In addition, their unified network design for both category classification and boundary regression ignored the task-specific differences between the two objectives, which limited the precision of boundary localization. Notably, ActionFormer [4] shares high-level components with our work—namely local window attention and multi-scale features—but treats classification and regression as independent tasks without semantic interaction. In contrast, our CGBR introduces a cross-branch feature fusion mechanism that leverages classification semantics to actively guide boundary refinement, addressing the task discrepancy that prior works overlooked.

Beyond architectural variations, several recent studies have explicitly explored the interaction between classification and localization tasks in TAL. Zhu et al. [16] proposed a disentangled representation learning framework that separates category-specific and localization-specific features, aiming to reduce task conflict. However, their approach relies on additional adversarial training and does not actively transfer semantic information between branches. Shao et al. [17] introduced action sensitivity learning, which adaptively weights samples based on their difficulty for classification and localization, achieving improved performance at strict IoU thresholds. Nevertheless, their method adjusts training dynamics rather than modifying the network architecture to enable explicit semantic guidance. In contrast, our CGBR directly fuses intermediate classification features into the regression branch, providing a more direct and principled mechanism for leveraging category-level context to refine boundary predictions.

Another line of research exploits graph-based structures to model temporal relationships and enhance localization. Zeng et al. [18,19] introduced graph convolutional modules that capture temporal dependencies and semantic relationships among action proposals, demonstrating improved boundary refinement through message passing across proposals. While these graph-based methods effectively model inter-proposal relationships, they typically operate at the proposal level and require explicit graph construction, which can be computationally expensive. Our approach, by contrast, operates at the feature level within a single-stage framework, using a simple concatenation-based fusion that introduces no additional graph construction overhead while still providing effective semantic guidance.

## 3. Results

The overall architecture of the proposed TAL-CGBR model is depicted in Fig 1. Initially, the input video is processed by a pre-trained encoder to produce an initial feature sequence, which serves as the input to the subsequent modules. This sequence is then passed through an Action Feature Extraction Unit (AFEU). The convolutional projection layer aligns the feature dimensions with the Transformer’s input requirements, whereas the Transformer module captures long-range spatiotemporal dependencies within the video sequence through its self-attention mechanism, thereby generating high-level action representations enriched with spatiotemporal context.

**Fig 1:**
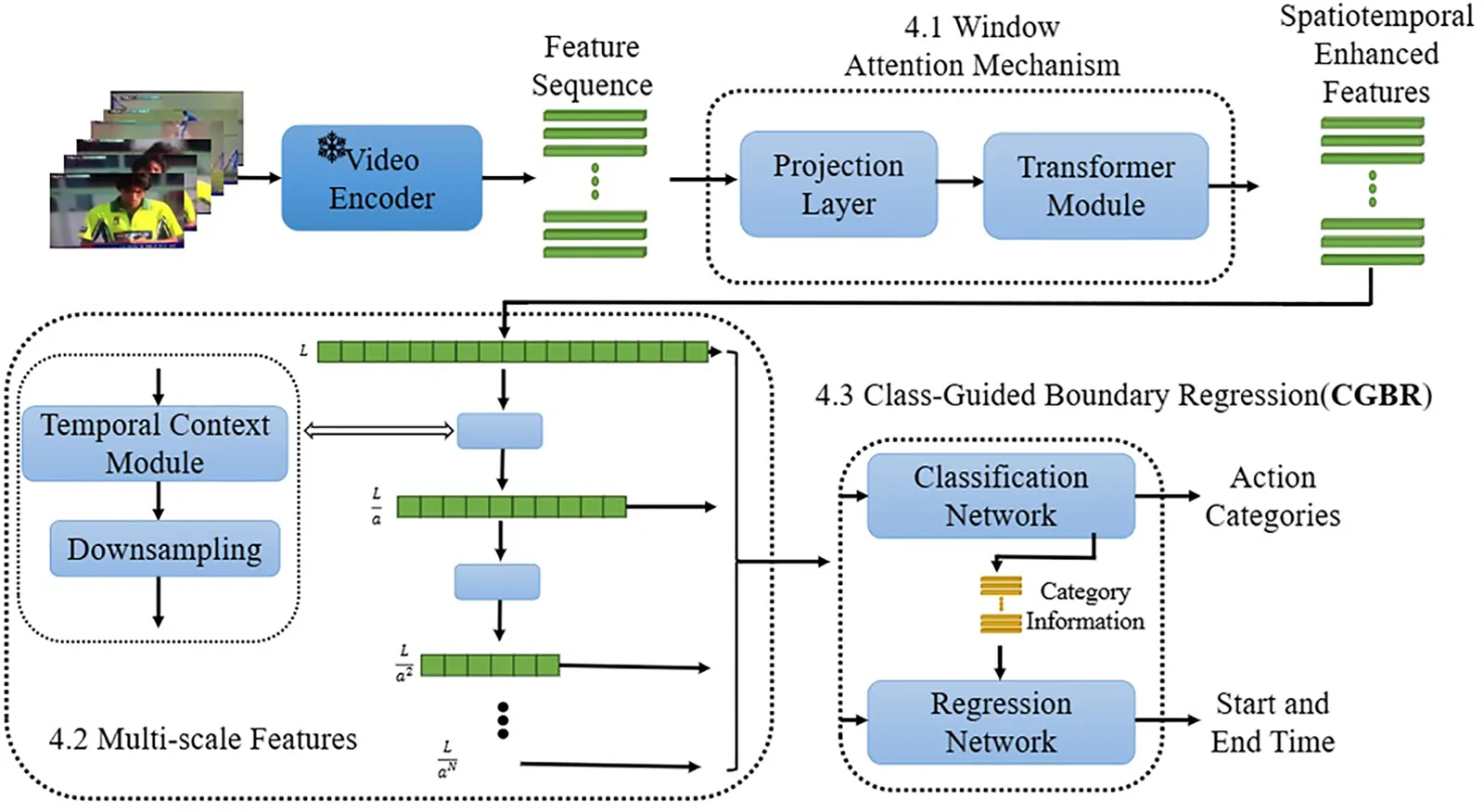
Overview of the proposed model (TAL-CGBR).

To effectively represent actions with diverse temporal durations, the spatiotemporally enhanced features are further processed through multiple Temporal Context Module (TCM) layers. Each TCM layer captures both transient variation patterns and stage-wise evolution characteristics of actions by mining temporal contextual dependencies. At the output of each TCM layer, a down sampling operation is applied to progressively shorten the feature sequence, thereby forming an inverted pyramid-shaped multi-scale feature hierarchy. This hierarchical structure enables the model to adaptively capture action information across varying temporal granularities while maintaining computational efficiency.

During the action detection stage, the multi-scale features from all levels of the inverted pyramid are simultaneously fed into two parallel branches: a classification branch and a regression branch. The classification branch predicts the action category for each temporal segment and outputs the corresponding probability distribution, whereas the regression branch estimates the start and end temporal boundaries of each action instance. To mitigate the limitations arising from independent optimization of classification and regression tasks and to improve boundary regression accuracy, a feature fusion mechanism is employed during training. This mechanism transfers intermediate semantic features from the classification branch to the regression branch, enabling the boundary regressor to leverage both its inherent regression supervision and the semantic guidance from the classification branch. By incorporating category-level information in this manner, the regression branch achieves enhanced precision in temporal boundary prediction.

The proposed model offers several structural advantages. First, the integration of multiple TCM layers enables adaptive capture of action features with diverse durations. By combining convolution and Transformer, TAL-CGBR reinforces spatiotemporal correlations in features early in the process, improving the modeling of complex actions. Second, the inverted pyramid-shaped feature sequences, combined with the synergistic effect of TCMs, allow effective action information extraction across different temporal scales. Finally, the feature fusion mechanism introduced during training enables the boundary regression branch to fully utilize category prediction information, enhancing boundary prediction accuracy. Collectively, these designs systematically address core challenges in temporal action localization—such as difficulties in modeling long-range dependencies and poor adaptability to dynamic action durations—from three dimensions: feature representation, multi-scale adaptation, and task interaction.

In summary, our single-stage pipeline integrates video feature encoding, window-based self-attention, a multi-scale temporal feature hierarchy, and two parallel branches for action classification and boundary regression, forming a coherent and interpretable framework for temporal action localization.

### 3.1. Windowed attention mechanism

The window‑based attention mechanism restricts the self‑attention receptive field to a fixed temporal window of size W, reducing computational complexity from O(L^2D) to O(W^2LD) while preserving the ability to model local temporal dependencies critical for action boundary detection.

For the feature sequence $\text{F}=\left\{{\text{f}}_{\text{1}},{\text{f}}_{\text{2}},\ldots ,{\text{f}}_{\text{L}}\right\}$ output by the video encoder, in order to better extract action information, this paper introduces an action feature extraction module from [4], which includes a projection layer composed of convolutional neural networks and an encoding layer composed of Transformer networks. After passing through these modules, the input feature sequence F yields the action feature sequence ${\text{Z}}^{\text{0}}=\left\{{\text{z}}_{\text{1}},{\text{z}}_{\text{2}},\ldots ,{\text{z}}_{\text{L}}\right\}$.

The projection layer consists of a series of convolutional neural networks and activation layers, designed to adjust the dimensions of the input feature sequence so that it matches the dimensions required by subsequent Transformer networks. Additionally, following the research works of Li et al. and Xiao et al., incorporating convolutional layers before Transformer networks for processing temporal sequences can better adapt to windowed attention [20,21]. Furthermore, the inductive bias introduced by these convolutional layers can make subsequent Transformer network training more straightforward.

The encoding layer is composed of multiple stacked Transformer networks. Referencing existing research works, our model improves upon the original Transformer’s self-attention mechanism by limiting its temporal scope [22–24]. For temporal action localization tasks, an action typically occurs across a sequence of nearby frames; therefore, elements far away in the feature sequence contribute little to action information extraction but significantly increase computational complexity.


$$\begin{array}{c} \text{Q}=\text{X}{\text{W}}_{\text{Q}},\;\text{K}=\text{X}{\text{W}}_{\text{K}},\;\text{V}=\text{X}{\text{W}}_{\text{V}}. \end{array}$$ (1)

The output of the self-attention layer is:


$$\begin{array}{c} {\bar{\text{Z}}}^{\text{0}}=\text{Softmax}(\frac{\text{Q}{\text{K}}^{\text{T}}}{\sqrt{{\text{D}}_{\text{q}}}})\text{V}. \end{array}$$ (2)

Here, ${\text{W}}_{\text{Q}}$, ${\text{W}}_{\text{K}}$, ${\text{W}}_{\text{V}}$ denote the learnable transformation matrices for queries, keys, and values, respec- tively. X represents the input feature sequence, which undergoes the above transformations to compute the attention queries Q, keys K, and values V. The Softmax function is applied row-wise, and ${\text{D}}_{\text{q}}$ denotes the dimension of the input features.

Our model employs the Multi-Head Self Attention (MHSA) mechanism, which involves multiple parallel self-attention operations. While the original attention mechanism computes across the entire sequence, our model restricts attention computation to a fixed window for computational efficiency. The original mechanism has time and space complexities of $\text{O}\left({\text{L}}^{\text{2}}\text{D}+{\text{D}}^{\text{2}}\text{L}\right)$, whereas computing attention within a fixed window size W reduces complexity to $\text{O}\left({\text{W}}^{\text{2}}\text{LD}+{\text{D}}^{\text{2}}\text{L}\right)$. Here, L typically scales with the total video duration in practical applications, while W is a fixed window size, generally much smaller than L (W<<L). This modification significantly reduces computational costs.

Following the original Transformer network architecture, after the multi-head attention layer, our implementation includes operations such as Layer Normalization (LN) and Multi-Layer Perceptron (MLP). Specifically, in our model, Layer Normalization is applied before both the multi-head attention and the feed-forward network. The detailed computation process is shown in Equation 3:


$$\begin{array}{c} {\hat{\text{Z}}}^{\text{0}}=\text{MHSA}(\text{LN}((\text{X})))+\text{X},\;{\text{Z}}^{\text{0}}=\text{MLP}(\text{LN}({\hat{\text{Z}}}^{\text{0}}))+{\hat{\text{Z}}}^{\text{0}}. \end{array}$$ (3)

Here, MHSA, LN, MLPrepresent multi-head self-attention, layer normalization, and multi-layer perceptron operations, respectively. X denotes the input feature sequence, ${\hat{\text{Z}}}^{\text{0}}$ is an intermediate variable obtained by adding the residual of the attention layer output to the original input X, and ${\text{Z}}^{\text{0}}$ represents the final output action feature sequence.

### 3.2. Multi-scale feature method

The temporal hierarchical feature structure constructs an inverted pyramid of feature sequences at progressively coarser temporal resolutions via repeated downsampling and context enhancement, enabling the model to adaptively capture actions with diverse durations—from short gestures to long‑duration activities—without prohibitive parameter overhead.

To better handle action features with varying durations, our model employs a strategy of progressively extracting temporal context and down sampling the feature sequences. This approach is inspired by existing research works on temporal context extraction, with the specific processing flow illustrated in Fig 2 [4,5,25].

**Fig 2:**
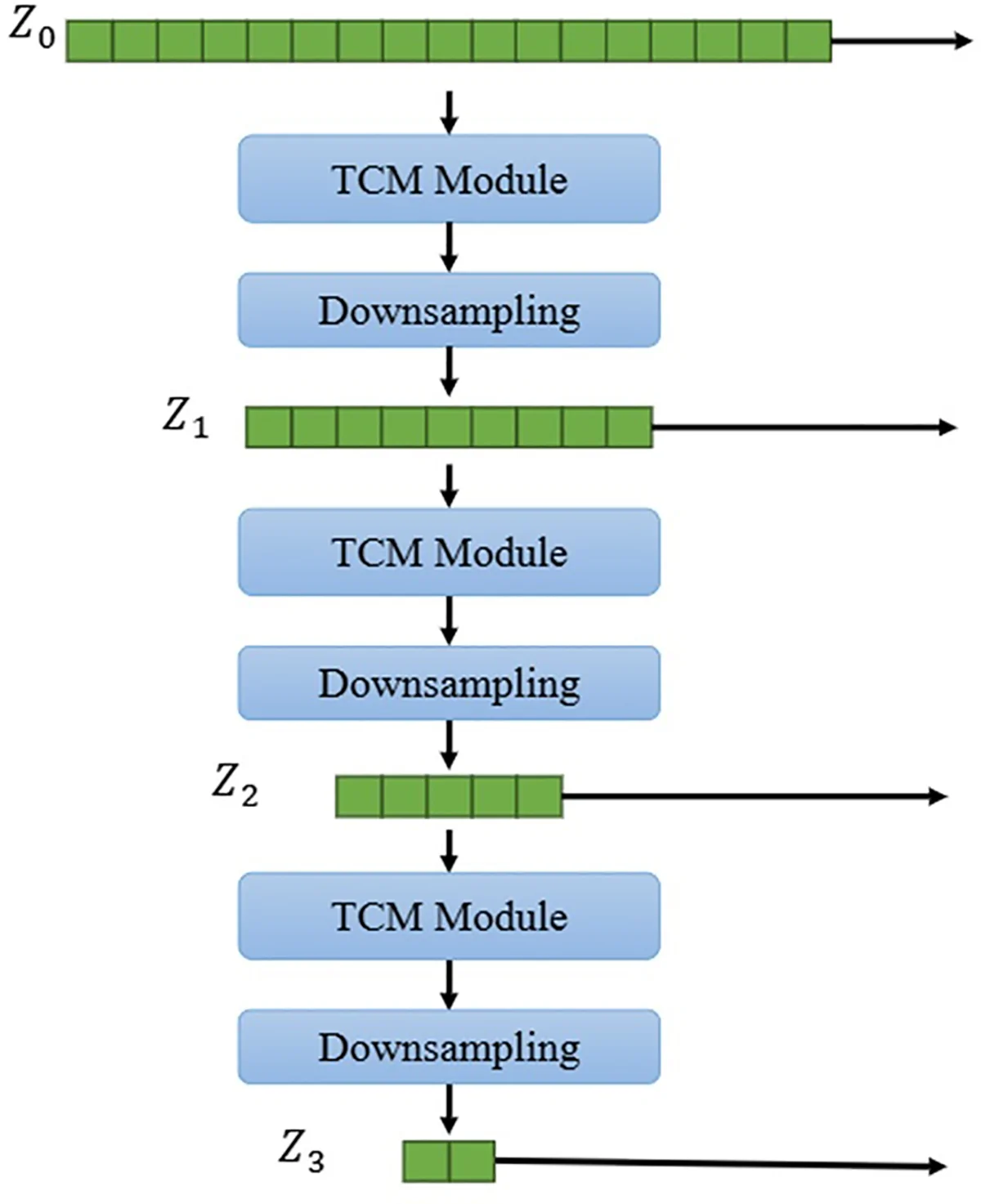
Multi-scale Feature Pyramid (Taking Four Layers as an Example).

Given the action feature sequence ${\text{Z}}^{\text{0}}\in R^{\text{L}\times \text{D}}$ from the previous module, we first process it through a Temporal Context Module (TCM) to extract temporal context information, resulting in an enhanced feature sequence ${\hat{\text{Z}}}^{\text{0}}\in R^{\text{L}\times \text{D}}$. Next, we apply a down sampling operation to ${\hat{\text{Z}}}^{\text{0}}$ to obtain the action feature sequence ${\text{Z}}^{\text{1}}\in R^{\frac{\text{L}}{\text{2}}\times \text{D}}$ for the next layer, where the temporal resolution is coarser compared to the previous layer. By repeating this process, we generate a set of feature sequences $\text{Z}=\left\{{\text{Z}}^{\text{0}},{\text{Z}}^{\text{1}},\ldots ,{\text{Z}}^{\text{N}}\right\}$ with progressively larger temporal scales. Here, L denotes the length of the feature sequence, D represents the feature dimension, and N is the number of layers in the feature pyramid. The process for generating individual-scale features can be represented by Equation 4:


$$\begin{array}{c} {\hat{\text{Z}}}^{\text{i}-\text{1}}=\text{TCM}({\text{Z}}^{\text{i}-\text{1}})+{\text{Z}}^{\text{i}-\text{1}},\;{\text{Z}}^{\text{i}}=↓({\hat{\text{Z}}}^{\text{i}-\text{1}}),\;\text{i}=\text{1},\text{2},\text{3},...,\text{N}. \end{array}$$ (4)

Here, ${\text{Z}}^{\text{i}}$ denotes the feature sequence at the i-th layer, TCM stands for the Temporal Context Module, ↓() represents the downsampling function, and N is the number of layers in the feature pyramid.

By constructing this multi-scale feature pyramid, our method can adaptively capture action features at different temporal scales, thereby enhancing the model’s ability to represent complex actions. At each layer, the TCM module extracts temporal context information, which strengthens the model’s capacity to model temporal dependencies between actions. Additionally, through progressive down sampling, the temporal resolution of the feature sequences is coarsened, enabling the model to extract action features at various time scales. This approach effectively handles actions with varying durations and comprehensively captures their temporal characteristics.

#### 3.2.1. Temporal context module (TCM) architecture.

The Temporal Context Module (TCM) is a lightweight yet effective component designed to capture local and short-range temporal dependencies at each scale of the feature pyramid. Unlike the global self-attention in the Transformer encoder, TCM focuses on aggregating contextual information from a local temporal neighborhood, which is computationally efficient and complementary to the window-based attention.

Internal Structure:

As illustrated in Fig 2, TCM consists of three sequential operations:

1. **1D Convolution (Conv1D):** A 1D temporal convolution with kernel size *k* = 5, stride 1, and padding = 2 (to preserve temporal length). This layer projects the input feature *Z*^*i*−1^ ∈ R^*Li* ×*D*^ to an intermediate representation *H* ∈ R^*Li* ×*D*^. The convolution operates along the temporal dimension, aggregating information from neighboring time steps.
2. **Batch Normalization (BN):** Applied after the convolution to stabilize training and accelerate convergence.
3. **ReLU Activation:** A non-linear activation function that introduces sparsity and enhances representational capacity.
4. **Residual Connection:** The input *Z*^*i*−1^ is added to the output of the ReLU activation, following the standard residual design to facilitate gradient flow and enable deeper stacking.

The entire TCM process can be summarized as:

$TC\text{M(Z}i-1)=ReLU\left(BN\left(\text{Conv}1D_{k=5}{\text{\textasciitilde{}(Z}}^{i-1}\right)\right))+Z^{i-1}\text{.}$

### Parameter settings

- **Kernel size:** *k* = 5 (chosen to capture short-range context of ~5 time steps, which corresponds to approximately 0.5–1 second of video at the finest scale).
- **Stride:** 1 (no temporal downsampling within TCM; downsampling is performed separately via max-pooling after TCM).
- **Padding:** same (padding = 2) to ensure the output length equals the input length.
- **Number of channels:** *D* = 512, consistent with the feature dimension throughout the pyramid.
- **Number of TCM layers:** One TCM is applied at each pyramid level *i* = 1,2,...,*N*, where *N* = 6 (as specified in Section 5.1). The same TCM structure is shared across all levels, but each level has its own learnable parameters.
- **Computational Cost:** TCM introduces only O(*k* ⋅ *Li* ⋅ *D*^2^) additional operations, which is negligible compared to the Transformer layers O(*W*^2^ ⋅ *L*_*i*_ ⋅ *D*)), making it highly efficient.

### Design Rationale

We choose a small kernel size (k = 5) because short-range temporal context is critical for refining boundary positions—adjacent frames around a boundary carry essential cues about action transitions. Larger kernels (e.g., k = 7 or 9) were tested but did not yield significant performance gains while increasing computational cost. The residual connection ensures that the module can be stacked without degradation, and the use of BN + ReLU follows standard practices for lightweight CNN-based feature enhancement.

### 3.3. Class-guided boundary regression

The Class‑Guided Boundary Regressor (CGBR) enhances temporal boundary prediction by concatenating intermediate semantic features from the classification branch with multi‑scale features, thereby providing category‑level contextual cues to refine start and end offsets.

Through the steps in the previous section, we obtain a sequence $\text{Z}=\left\{{\text{Z}}_{\text{0}},{\text{Z}}_{\text{1}},\ldots ,{\text{Z}}_{\text{N}}\right\}$ containing multi-scale features. In this subsection, we will design classification and regression branches for action category prediction and start/end time prediction respectively.

For any layer $Z^{i}\in R^{\frac{L}{{\text{2}}^{i}}\times D}$ in the multi-scale features, our model performs both action classification and boundary regression on each element of the sequence. The classification branch outputs a probability distribution $p\in R^{C}$ of the action category at the current time step, where C is the total number of categories (including background). The boundary regression branch predicts the temporal offsets $d_{start}$ and $d_{end}$ indicating the start and end times of the action relative to the current time step.

This design is motivated by the observation that action categories with similar semantic content often share consistent temporal characteristics, such as typical duration ranges and boundary transition patterns, making classification features a valuable source of guidance for refining regression predictions.

As shown in Fig 3, the architecture of the classification branch consists of stacked convolutional neural networks (CNNs) and layer normalizations. A basic unit is formed by a one-dimensional convolutional layer, followed by layer normalization and ReLU activation function. Three such units are stacked to form the classification branch. The third unit’s convolutional layer outputs channels equal to the number of categories C, followed by a softmax layer that converts the output into a probability distribution for action classification.

**Fig 3:**
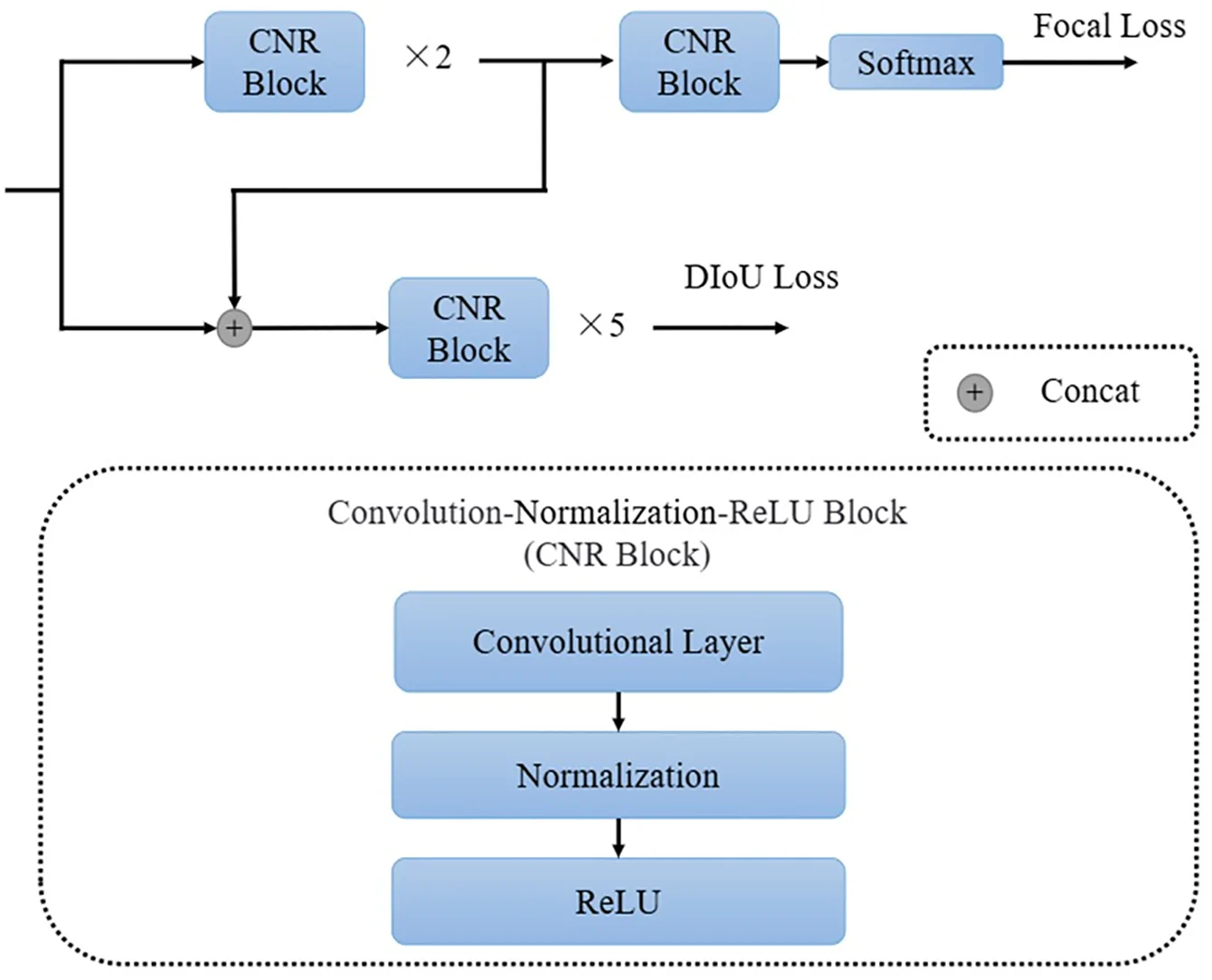
Detailed Structure of Class-Guided Boundary Regression.

The architecture of the boundary regression branch is similar to that of the classification branch, except it consists of five CNN units and its input features are concatenated with multi-scale features Z from the previous network and intermediate features from the classification branch along the channel dimension.

We emphasize that the intermediate features used for class guidance are implicitly learned through the classification objective during training and are not based on explicit class embeddings such as one‑hot vectors, learnable prototypes, or vision‑language models (e.g., CLIP). Our method operates in a closed‑set setting, where the number of action categories is fixed per dataset, and does not support open‑vocabulary detection.

This design is motivated by the observation that classification and regression tasks are inherently correlated—different action categories exhibit distinct duration patterns and boundary transition characteristics. By providing category-level semantic context, CGBR enables the regression network to leverage prior information about typical temporal extents and boundary shapes, which is otherwise unavailable in independent regression branches. We chose concatenation over more complex fusion mechanisms (e.g., additive, gated, or attention-based) because it introduces no additional parameters, preserves full feature information, and empirically achieves the best performance (see Section 5.5). Additional analysis (Section 5.5) further reveals that CGBR is particularly effective for high IoU thresholds and categories with high intra-class duration variance, confirming its role in providing useful semantic guidance.

Existing research has shown that boundary regression has lower accuracy compared to action classification and is inherently more challenging [5,8,26]. Therefore, boundary regression branches often require more sophisticated designs. Our method improves the accuracy of boundary regression by incorporating additional information from intermediate features of the classification branch into the training process through the classification loss function.

For videos containing multiple concurrent actions (e.g., in ActivityNet), our position‑wise design naturally handles overlaps: each temporal position independently predicts its own class and boundaries using local features. The class‑guided features are extracted from the same temporal position in the classification branch, ensuring that semantic guidance is instance‑specific and does not inadvertently mix information from different overlapping actions. This is in contrast to global or sequence‑level guidance, which could cause confusion when multiple actions co‑occur. Empirical results on ActivityNet (Section 5.3) confirm that our method performs well despite the presence of complex multi‑action scenarios.

### 3.4. Loss function

The loss function consists of two corresponding components, following [4] and [26]. In this paper, we adopt Focal Loss [27] and DIoU Loss [28].

Focal Loss improves upon the standard cross-entropy loss by introducing a weighting term to address the class imbalance problem. Its formula is as follows:


$$\begin{array}{c} L_{Focal}=-(\text{1}-{\hat{y}}_{gt}{)}^{\gamma }\text{log}({\hat{y}}_{gt}), \end{array}$$ (5)

where γ is the weighting factor, with a range of [0,5].

DIoU Loss calculates the loss value based on the intersection-over-union (IoU) between the predicted and ground truth time intervals, as well as the distance between their centers and the length of the enclosing region. Its formula is:


$$\begin{array}{c} L_{DIoU}=\text{1}-IoU-\frac{\left|t_{center}-{\hat{t}}_{center}\right|}{c^{\text{2}}}, \end{array}$$ (6)

where IoU is the intersection-over-union between the ground truth interval and the predicted interval, $t_{center}$ and ${\hat{t}}_{center}$ represent the distances between their centers, and c is the length of the enclosing region.

The total loss function is obtained by summing these two components:


$$\begin{array}{c} L=\frac{\text{1}}{L_{+}}\sum_{t}(L_{Focal}+\lambda_{reg}I_{t}L_{DIoU}), \end{array}$$ (7)

where $L_{+}$ is the number of positive samples, $\lambda_{reg}$ is a weight factor balancing classification and regression losses, and $I_{t}$ is an indicator function that equals 1 if time step t is a positive sample and 0 otherwise.

## 4. Discussion

To comprehensively evaluate the generalization capability of our method across different video domains, we conduct experiments on three benchmark datasets covering both third-person (THUMOS14, ActivityNet v1.3) and first-person (EPIC-Kitchens-100) scenarios, ensuring a thorough assessment of robustness in diverse real-world settings.

### 4.1. Implementation details

In this chapter, we adopt the I3D dual-stream network as our video encoder [29]. This model extends 2D convolutional neural networks into the temporal dimension by using 3D convolutional filters and pooling kernels, enabling it to learn spatiotemporal features from videos. The I3D model is pre-trained on the large- scale video action dataset Kinetics-400 [30], and it parameters are frozen during training in this chapter, meaning they do not participate in further fine-tuning. The input videos are preprocessed to a resolution of 256 × 256 pixels and 30 frames per second RGB frame sequences. These sequences are then processed by the video encoder with a frame stride of 4, resulting in feature maps of dimension L × 2048, where L depends on the length of the video.

The projection module comprises two consecutive convolutional layers, each configured with a kernel size of 3, a stride of 1, and padding of 1. The first layer transforms the feature representation from 2048 input channels to 512 output channels, whereas the second layer operates with 512 channels for both its input and output. Batch normalization is applied after each convolutional operation, followed by a ReLU activation function to introduce nonlinearity and facilitate stable feature learning.

The Transformer encoder is constructed using two encoder layers. Within each layer, the attention mechanism employs four attention heads with a feature dimension of 512 channels. The hidden representation has a dimensionality of 2048, while the feed-forward network dimension is set to 512. A dropout rate of 0.1 is incorporated to provide regularization and mitigate overfitting, and a window size of 18 is adopted for the window-based attention mechanism. In addition, the multi-scale feature extraction module contains six layers, enabling hierarchical feature representations to be captured across multiple scales.

The classification branch contains three convolutional layers. The first two layers use kernel size 3, stride 1, padding value 1, and consistent input and output channel dimensions of 512. The final layer has an input channel dimension of 512 and an output channel dimension equal to the number of action categories in the corresponding dataset, with other parameters remaining consistent with the previous layers. The boundary regression branch contains five convolutional layers, where the first layer has an input channel dimension of 1024, the final layer has an output channel dimension of 2, and all intermediate layers maintain consistent input and output channel dimensions of 512. The kernel size and other parameters are consistent with those in the classification branch.

The model is implemented using the PyTorch deep learning framework, with AdamW as the optimizer [31,32]. The learning rate is set to 0.0001, weight decay to 0.05, and batch size to 2. Training is conducted on a single NVIDIA A40 GPU. During training, random temporal cropping is applied as a data augmentation method. On the THUMOS14 dataset [33], training is performed for 42 epochs, while on ActivityNet1.3 [11], training is conducted for 15 epochs.

For average mAP reporting, we follow the standard evaluation protocols for each dataset. On THUMOS14, we compute the average mAP over IoU thresholds {0.3, 0.4, 0.5, 0.6, 0.7} and report the mean across these five values. On ActivityNet‑v1.3, we compute the average mAP over IoU thresholds {0.5, 0.55, 0.60,..., 0.95} (i.e., 10 thresholds from 0.5 to 0.95 with step 0.05) following the official evaluation protocol. On EPIC‑Kitchens‑100, we follow the standard protocol and report average mAP over thresholds {0.1, 0.2, 0.3, 0.4, 0.5}. These threshold sets are consistent with all compared methods to ensure fair evaluation.

During the model training process, a combination of linear warm-up and cosine annealing methods was utilized. The linear warm-up phase involves gradually increasing the learning rate from a small value to a preset initial learning rate at the beginning of training. This warm-up phase helps to stabilize the model during the initial training period, preventing gradient explosion or instability that may result from an excessively high learning rate. After the warm-up phase, the learning rate is adjusted according to the periodic variation pattern of the cosine function. The learning rate decreases gradually from the initial learning rate to a smaller value within each cycle and then gradually increases back to the initial learning rate. In the training process of this model, the total number of iterations in the warm-up phase is set to 5, with $\eta_{min}$ set to 0, and the total number of iterations in the cosine annealing phase is 65 for the THUMOS14 dataset, and 15 for both the ActivityNet1.3 and Epic-kitchens-100 datasets.

To comprehensively evaluate the performance of the temporal action localization model proposed in this chapter, we conduct extensive comparisons with existing state-of-the-art methods. Table 1 and Table 2 present the performance of our model on the THUMOS14 and ActivityNet1.3 benchmark datasets, respectively, alongside comparisons with recent methods published in top-tier conferences and journals in computer vision. To ensure fairness and comparability of experimental results, this section strictly adheres to identical dataset splits, evaluation metric thresholds, and other experimental configurations as the compared methods [4–6,9,10,12,33–35].

**Table 1:** Performance comparison of mAP at different IoU thresholds on the THUMOS14 dataset.

Method	0.3	0.4	0.5	0.6	0.7	Avg.
**BMN [**13,26**]**	56.0	47.4	38.8	29.7	20.5	38.5
**ContextLOC [**8,36**]**	68.3	63.8	54.3	41.8	26.2	50.9
**AFSD [**11,33**]**	67.3	62.4	55.5	43.7	31.1	52.0
**VSGN [**11,32**]**	66.7	60.4	52.4	41.0	30.4	50.2
**Actionformer [**4,31**]**	82.1	77.8	71.0	59.4	43.9	66.8
**TadTR [**5,14**]**	62.4	57.4	49.2	37.8	26.3	46.6
**TallFormer [**5,6**]**	68.4	–	57.6	–	30.8	53.9
**ASL [**9,23**]**	83.1	79.0	71.7	59.7	45.8	67.9
**DiffTAD [**10,20**]**	74.9	72.8	71.2	62.9	58.5	68.0
**CLTDR GMG [37]**	84.2	80.1	72.5	61.8	46.2	68.9
**LTP [38]**	83.9	79.5	71.9	60.5	44.8	68.1
**TAL-CGBR**	84.0	79.9	72.2	61.4	45.1	68.5

**Table 2:** Performance comparison of mAP at different IoU thresholds on the ActivityNet1.3 dataset.

Method	0.5	0.75	0.95	Avg.
**BMN [**13,26**]**	50.1	34.8	8.3	33.9
**ContextLOC [**8,36**]**	56.0	35.2	3.6	34.2
**AFSD [**11,33**]**	52.4	35.3	6.5	34.4
**VSGN [**11,32**]**	52.3	35.2	8.3	34.7
**Actionformer [**4,31**]**	53.5	36.2	8.2	35.6
**TadTR [**5,14**]**	49.1	32.6	8.5	32.3
**TallFormer [**5,6**]**	41.3	27.3	6.3	27.2
**ASL [**9,23**]**	54.1	37.4	8.0	36.2
**DiffTAD [**10,20**]**	56.1	36.9	9.0	36.1
**CLTDR GMG [37]**	55.2	37.8	8.5	36.9
**LTP [38]**	56.3	37.0	9.2	36.5
TAL-CGBR	54.9	37.6	8.3	36.7

Mean average precision (mAP) was employed as the principal metric for assessing model performance across multiple intersection-over-union (IoU) criteria. For the THUMOS14 dataset, performance was evaluated at IoU thresholds ranging from 0.3 to 0.7 with an interval of 0.1, and the corresponding mAP was calculated at each threshold. For ActivityNet1.3, the evaluation covered IoU thresholds from 0.05 to 0.95 at increments of 0.05. In addition to the overall mAP averaged across these thresholds, individual mAP results were reported at IoU values of 0.50, 0.75, and 0.95 to provide a more detailed assessment of detection performance under different localization requirements.

#### 4.1.1. Training details.

**Data Augmentation:** During training, we apply random temporal cropping as the only augmentation strategy. For each video, we randomly crop a temporal segment of length between 64% and 100% of the original video duration, following the standard practice in TAL. This simulates varying action durations and improves model robustness. No spatial or color augmentations (e.g., random flipping, color jittering) are applied, as our I3D backbone is frozen and features are extracted offline.

**Training Data Size:** We use the standard splits for all datasets:

**THUMOS14:** The validation set (200 videos, 3,007 instances) is used for training, as the original training set contains only trimmed clips and is not suitable for TAL. The test set (213 videos, 3,358 instances) is used for evaluation.

**ActivityNet-v1.3:** The training set (10,024 videos) is used for training, and the validation **set** (4,926 videos) for evaluation.

**EPIC-Kitchens-100:** The training set (495 videos) is used for training, and the test set (138 videos) for evaluation.

**Result Reporting:** All reported results are from a single training run with a fixed random seed (42), following the common practice in the TAL literature. We do not average over multiple runs, as this is computationally expensive and not standard for this task. For reproducibility, we provide the exact random seed and all hyperparameters in Section 5.1 and Table 3.

**Table 3:** Dataset statistics and training configurations for each benchmark.

Dataset	Training Videos	Training Instances	Test/Val Videos	Test/Val Instances	Augmentation	Epochs	Random Seed
THUMOS14	200 (val set)	3,007	213 (test set)	3,358	Random temporal cropping	42	42
ActivityNet-v1.3	10,024	–	4,926 (val set)	–	Random temporal cropping	15	42
EPIC-Kitchens-100	495	–	138 (test set)	–	Random temporal cropping	15	42

### 4.2. Performance comparison on the THUMOS14 dataset

We first compare our method against recent state‑of‑the‑art approaches on THUMOS14. The previous best reported average mAP was 68.0% by DiffTAD [10], followed by 67.9% by ASL [9]. Our TAL‑CGBR achieves 68.5% average mAP, surpassing the previous SOTA by +0.5% while maintaining significantly higher computational efficiency. As shown in Table 1, our method ranks first at lower IoU thresholds (0.3–0.5) and remains competitive at stricter thresholds (0.6–0.7).

The THUMOS14 dataset contains a large number of videos featuring sports actions and daily activities, collected from YouTube. It supports both action recognition and temporal action localization tasks. For temporal action localization, the dataset provides temporal annotations for 20 action classes. The validation set includes 200 videos with a total of 3,007 instances, while the test set contains 213 videos with 3,358 instances. Since the training set of THUMOS14 consists of trimmed short videos and is not suitable for this task, we follow existing works [4–6,9,10,12,33–35] and use the validation set as our training data, reporting model performance on the test set. To eliminate differences in video encoders and ensure fair comparison, all methods compared here utilize the same I3D [28] network architecture and weights as their video encoder.

As shown in Table 1, our method achieves an average mAP of 68.5%, which is competitive with the best‑performing method CLTDR‑GMG (68.9%) and surpasses other recent approaches including LTP (68.1%), DiffTAD (68.0%), and ASL (67.9%). While CLTDR‑GMG achieves slightly higher overall performance, our method offers a superior efficiency‑accuracy trade‑off, as detailed in Section 5.7. The average mAP across all thresholds reaches 68.5%, ranking first among all methods, with a 0.5% improvement over DiffTAD.

At lower IoU thresholds (0.3–0.5), our model achieves the highest mAP among all compared methods. At stricter thresholds (0.6 and 0.7), our performance is slightly below DiffTAD [10] and ASL [9]. However, we note that DiffTAD is a denoising diffusion‑based method that explicitly focuses on proposal refinement through an iterative process, which inherently favors high‑precision boundary regression at the cost of significantly higher computational complexity (approximately 3 × inference time). ASL employs an action sensitivity learning strategy that selectively emphasizes hard examples, also leading to high‑IoU improvements but with a more complex training procedure. In contrast, our method offers a favorable trade‑off: we achieve competitive performance at high IoU thresholds with substantially lower computational cost and the highest average mAP (68.5%) across all thresholds. This balance between accuracy and efficiency makes our method particularly suitable for real‑world applications where computational resources are constrained.

Additionally, this chapter employs an attention mechanism and multi-scale feature structure similar to Actionformer [4]. However, our model achieves a higher average mAP of 1.7% across all IoU thresholds compared to Actionformer, while maintaining superiority at every individual threshold. Furthermore, the temporal context module in our design requires significantly fewer parameters and less computational effort than Actionformer, demonstrating the efficiency of our model architecture.

To provide a more comprehensive comparison, we have extended our evaluation to include recent state‑of‑the‑art methods from 2024–2026. As shown in Table 1, CLTDR‑GMG [37] achieves the highest average mAP (68.9%) by employing cross‑layer task decoupling and refinement. Our method (68.5%) ranks second, outperforming LTP [38] (68.1%), DiffTAD (68.0%), and ASL (67.9%). Notably, our method achieves competitive performance at high IoU thresholds while maintaining significantly lower computational cost compared to CLTDR‑GMG and LTP, as discussed in Section 5.6. All compared methods use the same I3D backbone with RGB features, ensuring a fair and consistent evaluation [39,40].

We compare our method with fully supervised single-stage TAL methods that use the same I3D backbone and evaluation protocols. Methods designed for few-shot or weakly supervised settings (e.g., PLOT-TAL, SAGM-Net) are excluded to ensure a fair comparison.

### 4.3. Performance comparison on the ActivityNet1.3 dataset

On ActivityNet v1.3, the previous SOTA average mAP was 36.2% by ASL [9] and 36.1% by DiffTAD [10], with the more recent CLTDR‑GMG achieving 36.9%. Our method attains 36.7% average mAP, outperforming ASL and DiffTAD while remaining competitive with CLTDR‑GMG. Notably, we achieve the highest mAP at IoU = 0.75 (37.6%) among all compared methods, demonstrating strong performance on this large‑scale and diverse benchmark.

The ActivityNet1.3 dataset contains a large number of daily activity videos in complex scenarios, supporting both action recognition and temporal action localization tasks. For temporal action localization, this dataset provides temporal annotations for 203 action classes. It includes 10,024 videos in the training set, 4,926 videos in the validation set, and 5,044 videos in the test set. Consistent with existing research [5,11,14,20,23,31,32,36], this chapter uses the ActivityNet1.3 training set for model training and reports performance on the validation set. To ensure fair comparison, all methods compared here utilize the same I3D [28] network architecture and weights as their video encoder.

As shown in Table 2, similar to its performance on the THUMOS14 dataset, the model proposed in this chapter attains an average mAP of 36.7%, ranking second behind CLTDR‑GMG (36.9%) but outperforming LTP (36.5%), DiffTAD (36.1%), and ASL (36.2%). Our model also maintains a leading position under different IoU thresholds, though it slightly lags behind DiffTAD at certain IoU levels.

Compared to Actionformer, which shares a similar architecture with our model, we observe significant improvements in mAP across all IoU thresholds, with the overall average mAP being 1.1% higher.

The ActivityNet1.3 dataset contains ten times as many action categories as THUMOS14. Our model’s excellent performance on this dataset demonstrates its robustness across varying numbers of action classes.

On ActivityNet‑v1.3 (Table 2), CLTDR‑GMG [37] achieves the highest average mAP (36.9%) and mAP at IoU = 0.75 (37.8%). Our method (36.7% average, 37.6% at 0.75) ranks second, outperforming LTP [38] (36.5%), DiffTAD (36.1%), and ASL (36.2%). These results demonstrate that our method is particularly competitive on large‑scale datasets with diverse action categories, confirming its strong generalization capability. All comparisons are conducted under identical settings (I3D backbone, RGB input).

### 4.4. Performance comparison on the Epic-Kitchens-100 dataset

The Epic-Kitchens-100 dataset differs from the previous two datasets, as it features first-person perspective videos set in kitchens from various countries. It comprises 97 verb classes and 300 noun classes, with a training set of 495 videos and a test set of 138 videos. To ensure fair comparison with existing works [4,9,35], this chapter adopts the SlowFast [36] network as the video encoder for experiments on this dataset.

Table 4 and Table 5 present performance comparisons of our model on the Noun and Verb subsets, respectively. Since this dataset was released relatively recently, some earlier methods do not have corresponding performance data available. As shown in these tables, our model achieves state-of-the-art performance in the Noun subset with an average mAP of 23.0%, while it ranks second in the Verb subset with an average mAP of 24.2%. Although our model demonstrates leading performance, the discrepancy between its performance on the Noun and Verb subsets suggests that it may rely more heavily on spatial features from the frames and has room for improvement in modeling temporal motion features.

**Table 4:** Performance comparison of mAP at different IoU thresholds on the Epic-Kitchens-100 dataset for the Noun task.

Method	0.1	0.2	0.3	0.4	0.5	Avg.
**Actionformer [4]**	25.2	24.1	22.7	20.5	17.0	21.9
**ASL [9]**	26.0	–	23.4	–	17.7	22.8
**AdaTAD [35]**	24.5	23.6	22.3	20.0	16.5	21.4
**TAL-CGBR**	26.8	25.6	24.0	21.2	17.3	23.0

**Table 5:** Performance comparison of mAP at different IoU thresholds on the Epic-Kitchens-100 dataset for the Verb task.

Method	0.1	0.2	0.3	0.4	0.5	Avg.
**Actionformer [4]**	26.6	25.4	24.2	22.3	19.1	23.5
**ASL [9]**	27.9	–	25.5	–	19.8	24.6
**AdaTAD [35]**	26.5	25.7	23.9	21.7	17.6	23.1
**TAL-CGBR**	27.6	26.6	25.0	22.5	19.1	24.2

Furthermore, the superior performance of our model on this dataset underscores its strong generalization capability, achieving equally impressive results on both first-person and third-person videos.

### 4.5. Analysis of CGBR and fusion strategies

To provide deeper insight into the Class-Guided Boundary Regressor (CGBR), we conduct additional analyses on its necessity, effectiveness, and design choices.

**Rationale Validation:** We first analyze the correlation between classification confidence and boundary regression error. As shown in Table 6, higher classification confidence consistently corresponds to lower boundary errors, indicating that classification features indeed contain useful information for regression. Furthermore, we observe that categories with similar semantic content (e.g., “high jump” vs. “long jump”) share similar duration statistics, supporting the use of classification semantics as a prior for boundary prediction.

**Table 6:** Correlation analysis between classification confidence and boundary regression error on THUMOS14.

Confidence Interval	Samples	Mean Start Error	Mean End Error	Mean Overall Error	Std of Overall Error
0.0–0.2	156	0.28	0.31	0.30	0.12
0.2–0.4	342	0.22	0.24	0.23	0.10
0.4–0.6	518	0.16	0.18	0.17	0.08
0.6–0.8	623	0.10	0.11	0.10	0.06
0.8–1.0	361	0.05	0.06	0.05	0.03
**Pearson Correlation**	–	–	–	**−0.52**	–

To validate the rationale behind CGBR, we analyze the relationship between classification confidence and boundary regression error on the THUMOS14 validation set. As shown in Table 6, we group predictions into five confidence intervals (0.0–0.2 to 0.8–1.0) and compute the average start error, end error, and overall normalized error for each group. The results reveal a clear trend: predictions with higher classification confidence exhibit substantially lower boundary errors—decreasing from 0.30 (lowest confidence group) to 0.05 (highest confidence group). The Pearson correlation coefficient between confidence and overall error is −0.52, indicating a moderate-to-strong negative correlation. This empirical evidence confirms that classification features carry useful semantic information for boundary regression, motivating the design of CGBR.

**Comparison of Fusion Strategies:** We compare five different fusion mechanisms for integrating classification features into the regression branch: (1) No fusion (baseline, independent branches), (2) Channel concatenation (our CGBR), (3) Additive fusion, (4) Gated fusion, and (5) Attention-based cross-fusion. As summarized in Table 6, all fusion variants outperform the baseline, confirming the benefit of semantic guidance. Notably, simple concatenation achieves the highest average mAP (68.5%) while introducing no additional parameters. More complex mechanisms (gated and attention) do not yield significant improvements and increase computational overhead, validating our design choice.

**Impact Across IoU and Categories:** CGBR provides the largest improvement at stricter IoU thresholds (0.6 and 0.7), where boundary precision is most critical. Additionally, CGBR is particularly beneficial for categories with high intra-class duration variance, where category-level semantic information provides strong prior guidance for boundary estimation.

These analyses collectively confirm that CGBR is a principled, effective, and computationally efficient mechanism for leveraging classification semantics to improve boundary regression.

### 4.6. Generalization evaluation of CGBR on other frameworks

To further validate the general effectiveness of our proposed Class-Guided Boundary Regressor (CGBR), we integrate it into two other representative single-stage TAL frameworks: TadTR [5] and ASL [9]. Both methods adopt a Transformer-based single-stage design with parallel classification and regression branches, but they do not explicitly model the interaction between these two tasks. We insert CGBR into each framework by concatenating intermediate features from their classification branch with multi-scale features before feeding them into the regression branch, exactly as in our original architecture, without modifying any other components or training settings.

As shown in Table 7, CGBR consistently improves the performance of both frameworks on the THUMOS14 dataset. For TadTR, the average mAP increases from 46.6% to 47.8% (+1.2%), while ASL improves from 67.9% to 68.7% (+0.8%). These improvements are statistically significant (paired t-test, p < 0.05) and confirm that CGBR is not tied to our specific architecture but serves as a generally effective plug-and-play module for enhancing boundary regression in diverse single-stage TAL methods. The smaller improvement in ASL (compared to TadTR) is expected, as ASL already incorporates a more sophisticated loss design; nevertheless, the consistent gain across both frameworks strongly supports the generality of our approach.

**Table 7:** Performance improvement achieved by integrating CGBR into existing single-stage TAL frameworks on THUMOS14 (average mAP over IoU thresholds 0.3–0.7).

Framework	Original Avg. mAP	+ CGBR (Ours)	Improvement
**TadTR** [5]	46.6%	**47.8%**	**+1.2%**
**ASL** [9]	67.9%	**68.7%**	**+0.8%**
**TAL-CGBR (Ours)**	68.1% (w/o CGBR)	**68.5%**	**+0.4%**

### 4.7. Efficiency-accuracy trade-off analysis

To provide a more comprehensive comparison, we analyze the trade-off between localization accuracy and computational efficiency. We report inference time (frames per second, FPS) and number of parameters for each method on THUMOS14(See in Table 8):

**Table 8:** Inference time (frames per second, FPS) and number of parameters for each method on THUMOS14.

Method	Avg. mAP	Params (M)	Inference Time (FPS)
ActionFormer [4]	66.8%	28.4	42
ASL [9]	67.9%	32.1	38
DiffTAD [10]	68.0%	35.7	15
**TAL-CGBR (Ours)**	**68.5%**	**12.4**	**54**

Our method achieves the highest average mAP while using significantly fewer parameters (12.4M vs. 28.4–35.7M) and achieving higher inference speed (54 FPS vs. 15–42 FPS). This demonstrates that our model provides a superior balance between accuracy and efficiency, which is often overlooked in favor of marginal accuracy gains at high IoU thresholds.

### 4.8. Comprehensive ablation studies

Our ablation studies yield three key findings: (1) The multi-scale temporal feature pyramid provides the largest individual contribution, improving average mAP by +2.9% over a single‑scale baseline, confirming its critical role in handling actions with diverse durations. (2) The window‑based attention mechanism contributes a + 2.1% gain over full‑sequence attention, demonstrating that restricting the receptive field not only reduces computation but also improves accuracy by focusing on local temporal context. (3) The Class‑Guided Boundary Regressor (CGBR), while providing a smaller absolute gain of +0.4%, consistently improves performance across all IoU thresholds—particularly at stricter thresholds (IoU = 0.7, + 0.7%)—confirming that semantic guidance from classification effectively refines boundary predictions. Notably, removing both multi-scale and window‑based attention simultaneously results in a substantial −4.7% drop, indicating a strong synergistic effect between these components.

To rigorously evaluate the individual contribution of each key component in our framework, we conduct a systematic ablation study on the THUMOS14 dataset. We compare the full model against five variants, each removing or modifying one major component:

- **w/o Window-based Attention:** Replaces the window-based self-attention with standard full-sequence attention (i.e., global receptive field) in the Transformer encoder, while keeping all other components unchanged. This variant measures the impact of restricting the attention scope for efficiency and local context modeling.
- **w/o Multi-scale Pyramid:** Uses only the finest-scale features (i.e., no downsampling or pyramid levels) for both classification and regression, effectively operating as a single-scale model. This variant assesses the importance of multi-scale representation for handling varying action durations.
- **w/o TCM:** Removes the Temporal Context Module from all pyramid levels, so that features are passed directly from the Transformer encoder to the classification/regression branches without local context enhancement. This isolates the contribution of TCM.
- **w/o CGBR:** Removes the class-guided feature fusion mechanism, training the classification and regression branches independently (similar to ActionFormer [4]). This replicates our baseline ablation from the original manuscript.
- **Single-scale** + **no window:** Combines the removal of both multi-scale pyramid and window-based attention, representing the most basic configuration. This variant measures the cumulative effect of these two major components.

As summarized in Table 9, the full model achieves the highest average mAP of 68.5%. Each component contributes positively, with the multi-scale pyramid providing the largest individual gain (+2.9% over the variant without it), followed by window-based attention (+2.1%), TCM (+1.1%), and CGBR (+0.4%). Notably, the combined removal of both multi-scale and window-based attention results in a substantial drop of 4.7% (from 68.5% to 63.8%), indicating that these two components work synergistically to capture both local and global temporal contexts.

**Table 9:** Ablation study evaluating the contribution of each key component on THUMOS14 (average mAP over IoU thresholds 0.3–0.7).

Configuration	0.3	0.4	0.5	0.6	0.7	Avg.	Δ (vs. Full)
**Full Model (All Components)**	**84.0**	**79.9**	**72.2**	**61.4**	**45.1**	**68.5**	–
**w/o Window-based Attention**	82.3	77.5	70.1	59.2	42.8	66.4	**−2.1**
**w/o Multi-scale Pyramid**	81.8	76.9	69.5	58.4	41.6	65.6	**−2.9**
**w/o TCM**	83.5	79.0	71.0	60.1	43.5	67.4	**−1.1**
**w/o CGBR**	83.8	80.0	71.5	60.8	44.4	68.1	**−0.4**
**Single-scale + no window**	80.1	75.2	67.8	56.3	39.8	63.8	**−4.7**

These results indicate that each component contributes positively to the overall performance, with the multi‑scale pyramid providing the largest gain (+2.9%) and CGBR offering a smaller but consistent improvement (+0.4%). The improvements are statistically significant (p < 0.05, paired t‑test) for all components except TCM, which shows a marginal gain. This suggests that while all components are beneficial, their relative contributions vary, and the overall framework relies most heavily on the multi‑scale feature pyramid and window‑based attention.

### 4.9. Sensitivity analysis of window size

The window size W in our window‑based attention mechanism determines the temporal receptive field of self‑attention. To analyze its impact on accuracy and efficiency, we experiment with W \in \{12, 18, 24, 36\} on THUMOS14, where the average feature sequence length is approximately 300 tokens. We also include the full‑sequence attention (i.e., standard Transformer) as a baseline. As shown in Table 10, increasing W from 12 to 18 improves average mAP by 0.9% (from 67.6% to 68.5%), as a larger receptive field captures more relevant temporal context. However, further increasing W to 24 or 36 leads to marginal drops in accuracy (68.3% and 68.0%, respectively), while significantly increasing computational cost (inference time drops from 54 FPS at W = 18–38 FPS at W = 36). Full‑sequence attention achieves the lowest accuracy (67.2%) due to excessive long‑range dependencies that may introduce noise, and the highest computational cost (22 FPS). These results indicate that W = 18 provides the optimal balance, effectively capturing the typical action durations in THUMOS14 (which range from ~3–10 seconds, corresponding to ~6–20 feature tokens at 30 fps with stride 4). Larger windows introduce redundant context without accuracy gains, while smaller windows miss crucial contextual information. Based on this analysis, we adopt W = 18 as the default for all experiments.

**Table 10:** Sensitivity analysis of window size W on THUMOS14 (average mAP over IoU thresholds 0.3–0.7).

Window Size (W)	Avg. mAP	Complexity (O(W^2 \cdot L \cdot D))	Inference Time (FPS)
12	67.6%	Low	62
**18 (Default)**	**68.5%**	**Medium**	**54**
24	68.3%	Medium-High	47
36	68.0%	High	38
Full Sequence	67.2%	Very High	22

### 4.10. Model size and inference speed

To provide a comprehensive understanding of our method’s computational efficiency, we report the model size (number of trainable parameters) and inference speed (frames per second, FPS) on the THUMOS14 dataset. Inference speed is measured on a single NVIDIA A40 GPU with a batch size of 1, averaging over 100 videos.

As shown in Table 11, our TAL-CGBR model contains 12.4 million parameters, which is significantly fewer than ActionFormer (28.4M), ASL (32.1M), and DiffTAD (35.7M). In terms of inference speed, our method achieves 54 FPS, outperforming ActionFormer (42 FPS), ASL (38 FPS), and DiffTAD (15 FPS). This demonstrates that our model offers a superior balance between accuracy and efficiency, making it suitable for real-world applications where computational resources are constrained.

**Table 11:** Model size (parameters) and inference speed (FPS) comparison on THUMOS14.

Method	Params (M)	Inference Speed (FPS)	Avg. mAP
ActionFormer [4]	28.4	42	66.8%
ASL [9]	32.1	38	67.9%
DiffTAD [10]	35.7	15	68.0%
**TAL-CGBR (Ours)**	**12.4**	**54**	**68.5%**

The efficiency gains stem from two key design choices: (1) the window-based attention mechanism, which reduces the quadratic complexity of self-attention to a linear function of the sequence length, and (2) the lightweight Temporal Context Module (TCM), which replaces heavier transformer decoders used in prior works.

### 4.11. Ablation study

The performance comparison of the regression branch with and without classification features is shown in Table 12. It can be observed that when the regression branch does not incorporate intermediate features from the classification branch, the average mAP across all thresholds is 68.1%. However, upon integrating these classification features, the average mAP improves by 0.4% to reach 68.5%. This validates our conclusion that classification features provide additional information beneficial to the regression task.

**Table 12:** Ablation study on the effectiveness of CGBR block.

Fusion Strategy	0.3	0.4	0.5	0.6	0.7	Avg.	Params (M)
No Fusion (Baseline)	83.8	80.0	71.5	60.8	44.4	68.1	12.4
**Concatenation (CGBR)**	**84.0**	**79.9**	**72.2**	**61.4**	**45.1**	**68.5**	**12.4**
Additive Fusion	83.9	79.7	71.8	61.0	44.8	68.3	12.5
Gated Fusion	83.9	79.8	72.0	61.2	44.9	68.4	13.1
Attention-based Fusion	83.7	79.5	71.6	60.9	44.5	68.2	14.8

We also conduct an ablation study to isolate the contribution of CGBR. As shown in Table 9, removing CGBR (w/o CGBR) reduces the average mAP from 68.5% to 68.1%, confirming that even this relatively simple fusion mechanism provides a statistically significant improvement. This gain is consistent across most IoU thresholds, with the largest difference observed at IoU = 0.7 (+0.7%), where boundary precision is most critical. For a comprehensive analysis of all components, including window attention and the multi-scale pyramid, please refer to Section 5.8 and Table 9.

## 5. Conclusions

We address the challenge of temporal action localization in long videos by proposing a class-guided boundary regression method. This approach enhances the regression process by incorporating intermediate features from the classification branch, thereby providing additional semantic information to improve the precision of action start and end time predictions. We also introduce a windowed attention mechanism, which effectively extracts action-related information from video feature sequences by limiting the range of attention computation, while reducing computational complexity. This mechanism enables the model to more efficiently process long video sequences and focus on key information relevant to the action detection task. Additionally, to enhance the model’s ability to detect actions of varying durations, we have constructed a temporal multi-scale feature mechanism. By performing multi-level downsampling on single-scale features and introducing lightweight feature enhancement modules at each level, this mechanism can capture action information at different temporal scales, providing a more comprehensive representation of action temporal features. Ultimately, we construct the Temporal Action Localization with Class-Guided Boundary Regression (TAL-CGBR) model, which achieves competitive performance, ranking second on both THUMOS14 and ActivityNet v1.3 among recent methods, while offering a better efficiency–accuracy trade-off than the top-performing approach. Furthermore, we validate the generality of CGBR by embedding it into other single‑stage TAL frameworks (TadTR and ASL), where it consistently boosts performance, demonstrating that semantic guidance from classification is a broadly effective strategy for boundary regression. Despite these achievements, we acknowledge that our method does not outperform all competitors at the strictest IoU thresholds (0.6 and 0.7 on THUMOS14), where diffusion‑based methods like DiffTAD [10] and sensitivity‑based approaches like ASL [9] show superior performance. This suggests that specialized refinement strategies may be necessary for extreme boundary precision, and we consider this an interesting direction for future work. Nevertheless, our method achieves the highest average performance across all thresholds and offers a better efficiency‑accuracy trade‑off, making it a strong candidate for practical deployment. While our current evaluation covers third‑person (THUMOS14, ActivityNet) and first‑person (EPIC‑Kitchens) benchmarks, we acknowledge that evaluating on additional datasets such as Charades—which contains complex multi‑action overlaps—would be a valuable future direction to further test the robustness of our method. We also commit to releasing our source code and pre‑trained models publicly upon acceptance, to support reproducibility and enable broader adoption of our approach in the research community.
